# Shift work and cognitive impairment in later life – results of a cross-sectional pilot study testing the feasibility of a large-scale epidemiologic investigation

**DOI:** 10.1186/s12889-018-6171-5

**Published:** 2018-11-14

**Authors:** Tobias Weinmann, Céline Vetter, Susanne Karch, Dennis Nowak, Katja Radon

**Affiliations:** 1 Institute and Clinic for Occupational, Social and Environmental Medicine, University Hospital, LMU Munich, Munich, Germany; 2 Munich Center of Health Sciences (MC-Health), Munich, Germany; 3 Department of Integrative Physiology, University of Colorado at Boulder, Boulder, CO USA; 4 Department of Psychiatry and Psychotherapy, Ludwig-Maximilian-University, Munich, Germany

**Keywords:** Shift work, Cognition, Cognitive dysfunction, Sleep disorders, Circadian rhythm, Social jetlag, Sleep, Aging

## Abstract

**Background:**

The effect of shift work on impairment of cognition in later life has not yet been sufficiently investigated. Therefore, we aimed at testing the feasibility of a large-scale epidemiologic study examining this putative association in a pilot study.

**Methods:**

Between January and April 2017, a cross-sectional study invited a random sample of 425 former and current employees of a German university hospital aged 55 years and older to undergo a cognitive test battery (including the Repeatable Battery for the Assessment of Neuropsychological Status, Trail Making Test, Letter-Number Span, and Vocabulary Test) and to complete a self-administered questionnaire on socio-demographic characteristics, chronotype, sleep, occupational history including shift work, and medical history. Fifty percent of the invitees were registered in the hospital’s occupational records as currently working or having worked in a shift system. The feasibility of a large-scale study was evaluated by the response of the study sample and the completeness of data. In addition, we calculated the prevalence of shift work and cognitive impairment in the study population.

**Results:**

Seventy five subjects (18%) completed the questionnaire, of whom 47 (11% of the total sample) participated in cognitive testing. In all but four items assessed in the questionnaire, the proportion of missing data was below 10 %, suggesting that the quality of collected data can be considered as high. Eighty percent of the participants reported that they ever worked in a shift system, indicating selective participation by exposure to shift work. With respect to chronotype, the majority of the study subjects rated themselves as rather evening type, while a quarter considered themselves as definite morning type. All cognitive tests could be carried out completely. We observed slight difficulties in at least one of the cognitive tests in 17 participants (36%) while two participants (4%) showed more pronounced signs of cognitive impairment.

**Conclusion:**

The present pilot study only partially supported the feasibility of the planned large-scale study. As response rates were low and depended on exposure to shift work, a better way of sampling and recruitment needs to be identified. The questionnaire and the test battery appear to be viable instruments.

**Electronic supplementary material:**

The online version of this article (10.1186/s12889-018-6171-5) contains supplementary material, which is available to authorized users.

## Background

With a global prevalence of about 24 million cases, neurodegenerative disorders such as Alzheimer’s disease (AD) are a major burden of illness in the modern world [1]. They not only cause severe suffering among patients and their families but also generate substantial costs for medical services, health insurances, and society as a whole. For instance, in the US alone the annual costs for health care and long-term care of AD patients are estimated at 259 billion US Dollars [2]. Given the ageing of the population especially in Western societies, this burden will further increase in the future [3]. However, despite the importance of this type of diseases and the urgency for action, it is so far not well understood which genetic, environmental, and occupational factors contribute to their aetiology [1, 4].

Recent studies indicate that the risk of developing neurodegenerative diseases might be associated with sleep disturbance and shift work, which is usually defined as work outside a regular daytime work schedule [5–9]. The proposed mechanism for this effect is circadian misalignment, which means that the circadian rhythm, i.e., the internal biological cycle that regulates sleep and wakefulness, is disturbed by an external stimulus such as shift work [10, 11]. Potential consequences include cancer, cardiovascular disease, diabetes, mental impairment, and neurologic as well as neurodegenerative disorders [7, 12–15]. With respect to potential underlying biological mechanisms, neuroinflammation due to sleep disturbances [16, 17], changes in the neuronal plasticity of the hippocampus [18], or impairment of melatonin production due to shift work could play a role – the latter as melatonin appears to contribute to the protection from neurodegenerative disease [19–21]. The plausibility of these mechanisms was shown in animal studies [22–24]. However, it has not yet been sufficiently investigated whether circadian misalignment has a causal influence on the development of neurodegenerative diseases such as AD [7].

The manifestation of AD includes different components, with cognitive symptoms being among the most important ones [3]. Even before a manifest diagnosis of AD can be made, most patients show mild forms of cognitive impairment as a precursor [2]. Thus, epidemiological studies investigating AD usually include subjects who show early forms of cognitive impairment and who are thus likely to develop a clinical diagnosis of AD in the future [25, 26]. So far, few studies examined the long-term effects of shift work on chronic impairment of cognition in later life yielding inconsistent results [6, 13, 14, 27]. In addition, from a methodological point of view, it is also interesting that studies including shift-workers or elderly individuals from the general population reported different experiences with respect to invitee’s willingness to take part in such studies. While prospective studies, for example the Nurses Health Study [27] or the French VISAT study [6], reported excellent response rates, other approaches such as cross-sectional studies in Australia and Norway could enrol only about a third of the invited individuals [25, 28]. When planning a study that aims to assess cognitive effects in current and former shift workers, it thus seems to be wise to first evaluate the feasibility of the planned design by means of a pilot study.

A major limitation of previous studies is the lack of adequate consideration of study subjects’ chronotype, i.e., their internal biological clock. Taking into account chronotype and sleeping habits, however, is crucial to determine circadian misalignment and potential health effects due to shift work [29–31]. For instance, results from studies on chronic disease such as diabetes or breast cancer point towards effect modification by chronotype [15, 32].

Therefore, we planned to design a large-scale epidemiologic study to investigate the putative association between shift work and cognitive impairment in later life with adequate consideration of individual sleeping habits and chronotype. Before implementing further steps, we evaluated the feasibility of the planned project by assessing study subjects’ willingness to participate, indication of selective participation, and the viability of the study instruments by means of the pilot study presented here.

## Methods

### Study design

We conducted a cross-sectional pilot study on a random sample of former and current employees of a German university hospital. For all study subjects, participation included completion of a study questionnaire and undergoing cognitive testing.

### Study sample and recruitment

From the hospital’s occupational register of all individuals that were employed within the last ten years prior to the study, the hospital’s administration identified all former and current hospital nurses and administrative staff aged 55 years and older as potential candidates for participation in the pilot study. Next, by random sampling, 500 of them were drawn to be invited to the pilot study. 250 of those were registered in the hospital’s occupational records as currently working or having worked in a shift system, while the remaining 250 individuals were employees with a regular daytime work schedule. 389 invitees (78%) were female. All potential study subjects were invited for participation in the pilot study via mail between January and April 2017. The mailed information consisted of an information sheet, an informed consent form, a paper-based study questionnaire, and a return envelope. Non-responders were reminded up to two times. The first reminder asked them to send back the documents they had received previously while the second reminder contained the full invitation package again (information sheet, informed consent form, questionnaire, return envelope). Those who gave written informed consent were then re-contacted and scheduled for an individual appointment for the cognitive assessment. A detailed description of the final sample is provided in the results section.

### Study questionnaire

The questionnaire assessed general and sociodemographic information (including age, sex, country of origin, marital status, formal education of the participant and his or her current or last partner, professional education, occupational history, height and weight), lifestyle factors (such as consumption of tobacco and alcohol, physical activity), and medical history (incl. Lifetime prevalence of neurologic, psychiatric, neurodegenerative, hormonal and endocrine disorders, cancer, asthma and allergies, sleep disturbances, and other major diseases).

To assess chronotype and sleeping habits, the questionnaire built upon the Munich Chronotype Questionnaire [33] and asked for sleeping patterns on free days (time of going to bed, time of falling asleep, minutes between going to bed and falling asleep, time of waking up, minutes between waking up and getting up, daytime napping (yes/no), and usage of an alarm clock (yes/no)) across lifespan (i.e., currently, at the age of 40 years, and at the age of 30 years). In addition, we asked for participants’ self-evaluation regarding their diurnal preference (definitive morning-type, rather morning-type, rather evening-type, definitive evening-type) using the respective question of the German version of the Morningness-Eveningness Questionnaire (MEQ) [34].

Moreover, we assessed study subjects’ occupational history including specific questions with respect to shift work exposure: shift work ever (yes/no), years working in shifts, years working in morning shifts, years working in night shifts, years working in morning shifts only, years working in night shifts only. In these questions, shift work was defined as tasks or jobs done outside regular daily working hours between 7 am and 6 pm.

As described above, participants received the questionnaire via mail and were asked to bring the complete questionnaire to their appointment for the cognitive test. On this occasion, the persons conducting the cognitive test checked the questionnaire for completeness and clarity and, if necessary, asked participants for additional information.

### Cognitive testing

To test participants‘cognitive abilities, a test battery consisting of the following tests was used: the Repeatable Battery for the Assessment of Neuropsychological Status (RBANS) [35], the German version of the Trail Making Test (TMT) [36], Letter-Number (LN) span [37], and the Vocabulary Test (Wortschatztest, WST) by Schmidt and Metzler [38].

The Repeatable Battery for the Assessment of Neuropsychological Status measured participants‘performance on five neurocognitive domains: immediate memory, visuospatial/constructional, language, attention, and delayed memory [35, 39]. For every participant, the raw scores were converted to an index score for each of the five domains by use of normative standards for the participants’ age group (50–59, 60–69, 70–79, and 80–89 years). The index scores were added to a sum of index scores, which was subsequently converted to a total scale index score, i.e. an age-standardised performance score.

The dyadic Trail Making Test assessed participants‘cognitive performance speed, speed of processing, visual search, scanning, mental flexibility, and executive functions [40]. Also for this test, raw scores were converted to age-standardised performance scores.

During the Letter-Number span, a series of random numbers and letters is orally presented to the participants. They are then asked to repeat first the numbers (from the smallest to the largest) and then the letters (in alphabetical order) [37]. By doing this, working memory, audible memory, and attention are measured. As no standards for this test exist, test scores were only used for clinical evaluation.

The Vocabulary Test measures verbal intelligence and speech comprehension estimating also premorbid intelligence and progression of dementia [38]. During this test, the participants completed 40 word recognition tasks. In each of them, they needed to identify the correct word out of a list containing the respective word and five distractors. After the test, raw scores were converted to standardised performance scores. As this test is independent of age, scores were not age-standardised.

In addition to calculation of raw scores and standardised performance scores, a trained psychologist clinically evaluated each participant’s performance in all four tests with respect to signs of cognitive impairment.

All tests were performed by two trained psychologists in a quiet test room at the university hospital from which the participants were recruited. The duration of the tests was about 45 to 60 min.

### Statistical analysis

The feasibility of the planned, large-scale study design was evaluated by i) study subjects’ willingness to participate (proportion of invited persons that agreed to fill out the questionnaire and to undergo cognitive testing), and indication of selective participation, and ii) completeness of data (number of missing data in the study questionnaire and proportion of cognitive tests that could be completed).

We present descriptive statistics of the participants’ socio-demographic characteristics using absolute numbers (n) and percentages (%) for the categorical variables and arithmetical mean, standard deviation (SD), minimum (Min), and maximum (Max) for the continuous variable age. The variables height and weight were converted to Body-Mass-Index (BMI) in kg/m^2^ before calculating the absolute and relative frequency of overweight participants (BMI ≥ 25 kg/m^2^). Moreover, we present lifetime prevalence of the disorders as well as summary statistics of sleep habits and chronotype.

We also report standardised test scores for all tests, namely the RBANS, TMT, and WST, as well as categorical outcomes, such as the proportion of participants who performed worse than average (score < 90), average (90–110) and above average (> 110). In addition, we calculated the absolute and relative frequency of participants with no, mild, or marked indication of cognitive impairment in the clinical evaluation. Chi-square test evaluated if those numbers differed statistically significantly between subjects with and without self-reported shift work history (alpha: .05).

## Results

Of the 500 randomly selected persons, 75 could not be contacted (70 had an invalid address, three persons had died, one person lived in a nursing home, another one in a hospice), resulting in a net sample of 425 potential participants. Of these, 75 subjects (18%) completed the questionnaire, of which 47 (11% of the total sample) participated in the cognitive testing. The remaining 28 persons either agreed to take part in the cognitive testing in the informed consent form, but could not be reached for scheduling, or did not appear for the test. Except for four variables (country of birth, time of falling asleep, minutes until falling asleep, time of waking up), the proportion of missing values for all variables in the questionnaire was less than 10 %.

### Summary statistics

The majority of the questionnaire respondents (87%) were female; mean age was 62 years. With regard to schooling, around three-quarters of the participants reported that they had a secondary modern school or high school diploma. In terms of occupational training, about half of the participants completed professional school. Sixty-eight percent of the participants were currently working. According to the calculated BMI, almost half of the subjects were overweight (Table 1). No participant indicated neurodegenerative diseases. However, 72% reported having suffered from sleep disorders, while 20% indicated mental disorders.

**Table 1:** Socio-demographic characteristics of questionnaire respondents (*N* = 75)

	Missing	Mean	SD^a^	Min	Max
Age (in years)	0	61.6	4.2	56	74
	Missing	N		%	
Gender	0				
Female		65		86.7	
Place of birth	17				
Germany		50		66.7	
Family status	0				
Single		15		20.0	
Married/partnership		42		56.0	
Divorced		10		13.3	
Widowed		8		10.7	
Schooling	0				
No degree		2		2.7	
Secondary modern school		20		26.7	
High school diploma		39		52.0	
University of applied sciences entrance qualification		3		4.0	
A level/Advanced Vocational Certificate of Education		6		8.0	
University degree		4		5.3	
Other		1		1.3	
Occupational training	0				
No degree		2		2.7	
Vocational/apprenticeship		25		33.3	
Professional school		39		52.0	
University degree		6		8.0	
Other		3		4.0	
Current occupation	0				
Full-time		30		40.0	
Part-time		21		28.0	
Pensioner		22		29.3	
Job-seeking		2		2.7	
Smoking	0				
Never smoked		40		53.3	
Previous smoker		21		28.0	
Current smoker		14		18.7	
Body-Mass-Index	3				
≥ 25 kg/m^2^		36		48.0	

^a^*SD* standard deviation

With respect to sleeping habits, subjects reported no significant changes in the time of bedtime or falling asleep between the ages of 30 and 40 and the current time. With regard to time of waking up, a slight tendency to earlier waking up at the present time can be seen in comparison to the earlier life periods (AM = 6:10 currently, 7:02 at 40, 7:16 at 30). Almost half of the respondents indicated to currently take a nap during the day while napping was reported much less frequently during the age of 40 (17%) and 30 years (15%).

At all three times, most of the participants rated themselves as rather evening type with the largest proportion of evening types at this point in time (36%). However, the proportion of self-assessed definitive evening type decreased with increasing age (24% at the age of 30 vs. 15% currently). Regarding the assessment as “rather morning type”, the trend was opposite with only 13% at the age of 30 compared to 24% at the current time (Table 2).

**Table 2:** MEQ classification of diurnal preference (*N* = 75)

		Definitive morning type		Rather morning type		Rather evening type		Definitive evening type	
	Missing	N	%	N	%	N	%	N	%
Present	3	16	21.3	18	24.0	27	36.0	11	14.7
At age 40 Years	6	20	26.7	11	14.7	23	30.7	15	20.0
At age 30 Years	6	19	25.3	10	13.3	22	29.3	18	24.0

60 participants (80%) indicated they had ever worked in shift work. Overall, they worked on average for 29 years in any shift system and for 24 years in a system including night shifts (Table 3).

**Table 3:** Self-reported cumulative exposure to shift work of questionnaire respondents who have ever worked in shift (*N* = 60)

	Missing	Mean	SD^a^	Min	Max
Years of shift work over lifetime					
Total years in shift work	6	28.9	13.2	0	46
Total years in night shift	1	24.2	13.6	0	46
Years in night shift only	1	2.95	9.3	0	45
Total years in morning shift	3	27.4	15.1	0	46
Years in morning shift only	2	2.22	6.4	0	35

^a^*SD* standard deviation

**Fig. 1:**
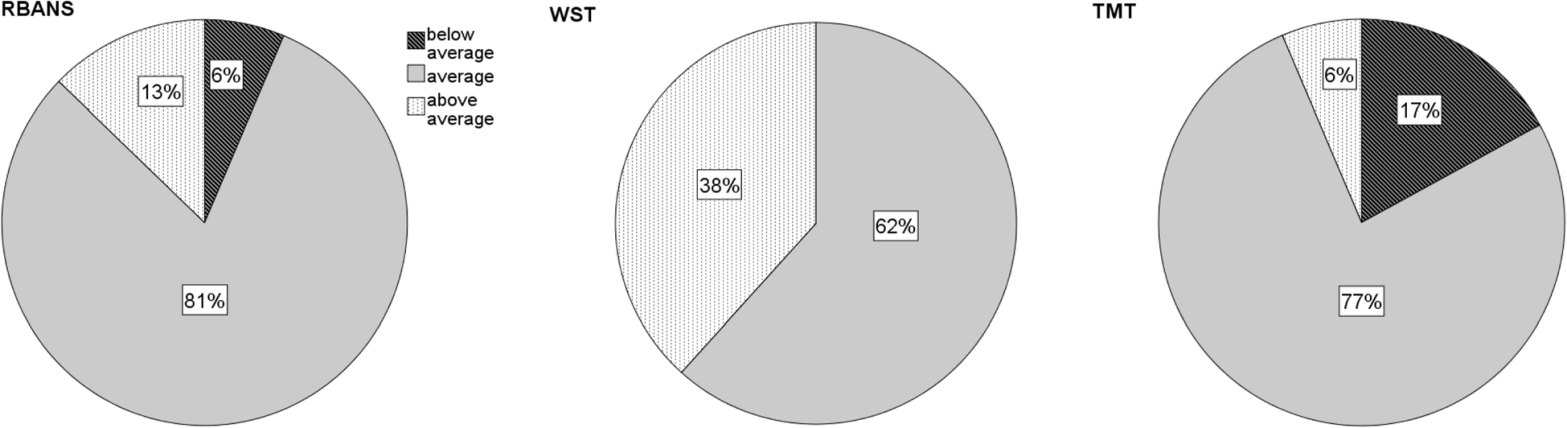
Relative frequencies of subjects (*N* = 47) with below average, average, or above average performance score for RBANS, Vocabulary Test (WST), and Trail Making Test (TMT)

Cognitive test scores were higher in the Vocabulary Test (AM = 110.2) than in the RBANS (AM = 102.1) or TMT (AM = 98.8) (Table 4). Accordingly, in the Vocabulary Test, none of the subjects tested scored below average, while 17% of participants in the TMT had a score below 90. However, in all three tests, the majority of subjects were in the average range (Fig. 1).

**Table 4:** Statistical characteristics of the test participants’ standardised cognitive test scores (*N* = 47) for the test methods RBANS, Vocabulary Test and Trail Making Test

	Missing	Mean	SD^a^	Min	Max
IQ					
RBANS	0	102.1	11.9	66	127
Vocabulary Test	0	110.2	10.4	86	129
Trail Making Test	0	98.8	9.3	57	130

^a^*SD* standard deviation

In the clinical evaluation of the individual overall picture from all four tests, 17 participants (36%) showed slight difficulties in at least one subscale of the cognitive tests. Clear signs of impairment were observed in two subjects (4%). The frequency of indication for slight or pronounced impairment did not differ between those working with (40%) and without shifts (40%; *p* = 0.97).

## Discussion

Our study aimed at examining the feasibility of a large-scale epidemiologic study to investigate the relationship between exposure to shift work and cognitive impairment in older age. The feasibility was evaluated based on invited subjects’ willingness to participate in the pilot study (response rate), indication of selective participation, and completeness of the collected data. While the willingness to participate was very low, especially among non-shift workers, the completeness of the data indicates that the survey instruments are very well applicable. Significant cognitive impairment was observed in very few subjects, but the test results indicated that around one third of the participants had at least slight difficulties with at least one cognitive test.

With respect to the response rate, only a very small proportion of the invited persons (11%) agreed both to participate in the cognitive testing and to complete the questionnaire. In addition, some invitees completed the questionnaire without participating in the testing. This clearly contradicts the feasibility of the proposed approach, since results that are not prone to considerable levels of selection bias require substantially greater willingness to participate. The fact that 80% of those who participated in the pilot study reported that they had worked in a shift system before, indicated that exposed individuals were more probable to take part in the study than non-exposed invitees were. Hence, there is a high likelihood of selection bias in our study population.

An explanation for the low response could be that it was too much effort for the invited persons to come to the examination centre and undergo a test procedure lasting one hour, especially without any financial incentive. The incentive to participate in a free screening of their cognitive abilities apparently did not suffice to convince the majority of the invitees to participate. At the same time, the examination of their cognitive performance could have led to a feeling of fear or shame in the participants, especially among those who felt to suffer some form of cognitive decline and among those who were still working. Beyond that, individuals with subjective decline even may have been concerned that the cognitive testing could have revealed indication for more severe impairment such as an early diagnosis of dementia which is sometimes associated with fear of discrimination or stigmatisation [41, 42]. This effect may have especially occurred as the aim of investigating cognitive impairment was explicitly mentioned and not encapsulated in a more general description. In that regard, our study was similar to an Australian pilot study with a related research question, which also suffered from a low response rate – although it was still higher than in our study [25]. The largest difference between our and the Australian study, however, was that they recruited a population-based sample while we conducted our study in an occupational setting. The fact that this setting was the one in which also the researchers are employed may have negatively influenced the invitees’ willingness to take part. There is also a tendency towards selective participation by sex as almost 90% of all participants were female (among all invitees, about 75% were females). Moreover, in another study using a test battery similar to ours and inviting individuals aged 35–74 years, willingness to participate was lowest in the oldest age group [43]. This suggests that it is generally difficult to recruit elderly people for psychometric testing. Apart from the potentially deterrent effect of the cognitive test battery, also additional reasons for the low response can be debated. One of them may be study design since, as already indicated in the introduction, shift work studies achieving high response rates seem to be predominantly prospective approaches. 

In contrast to the low willingness to participate, the quality of the collected data can be considered as high. All cognitive tests were completed and the proportion of missing information in the questionnaire was below 10 % for all but four variables. Thus, the procedure that participants first receive the questionnaire, fill it out at home and then bring it to the study centre where it is checked for missing or ambiguous information, can be regarded as highly feasible.

In terms of cognitive performance, in all three tests that were used to calculate a standardised performance score, at least 80% of all subjects tested had an average to above-average score. The Trail Making Test had the highest difficulty, with 17% of respondents performing substandard while the corresponding value for RBANS was 6 % and in the Vocabulary Test no participant had such a low test score. Accordingly, the clinical evaluation of the test results from all tests provided clear evidence of reduced cognitive performance in only two subjects. However, minor difficulties in at least one subscale were observed in about a third of the sample. This corresponds to the proportion of people with the syndrome *CIND* in the aforementioned Australian pilot study [25].

For the further planning of the project, selection and recruitment of the sample need to be improved considerably. First, it needs to be easier for subjects to participate in the cognitive testing. One way to do this within a retrospective cohort study could be to cooperate with the Occupational Health Service of a company that incorporates the testing into the regular occupational health check-ups. An alternative option could be a cross-sectional study recruiting participants via cooperating GPs that ask their patients to undergo the testing when they are in the GP’s surgery for medical examination. Another option could be a case-control study, which recruits cases with diagnosed mild cognitive impairment and a matched control group retrospectively assessing occupational history and chronotype of both groups. The largest potential limitation of a case-control design, however, is the retrospective exposure assessment, which in the case of our research question would be very prone to recall bias, especially among cases. Another means of possibly increasing the response rate might be to diminish the size of the cognitive test battery in order to reduce the expenditure of time for participation in the study. This would also reduce the time-wise and hence financial effort needed for field work on the part of the study team. The big drawback of shrinking the test battery, however, would be that less cognitive domains could be tested so that the assessment of participant’s cognition would be less comprehensive and thus potentially less reliable.

Regardless of the study design, subjects in the main study could be offered not only to receive their individual test results, but also other incentives that have been shown to be effective such as financial rewards or participation in a raffle [44, 45].

## Conclusions

In summary, the present pilot study only partially supported the feasibility of the planned large-scale study. The further planning in particular needs to focus on improving the recruitment of subjects to avoid selection bias. The study questionnaire and the cognitive test battery have been proven to be suitable.

## Additional files

Additional file 1: Study questionnaire. Study questionnaire in German. (PDF 115 kb)

Additional file 2: English translated version of the study questionnaire. (PDF 113 kb)

## Abbreviations

AD: Alzheimer’ disease
BMI: Body-Mass-Index
LN span: Letter-Number span
Max: Maximum
MEQ: Morningness-Eveningness Questionnaire
Min: Minimum
RBANS: Repeatable Battery for the Assessment of Neuropsychological Status
SD: Standard deviation
TMT: Trail Making Test
WST: Wortschatztest
