# Supplementary material for: Shift work and cognitive impairment in later life – results of a cross-sectional pilot study testing the feasibility of a large-scale epidemiologic investigation
Source: BMC Public Health. 2018 Nov 14;18:1256. doi: 10.1186/s12889-018-6171-5 (PMC6234599; doi:10.1186/s12889-018-6171-5)
Supplement: Supplementary file 1 — Study questionnaire. Study questionnaire in German. (PDF 115 kb) [file 12889_2018_6171_MOESM1_ESM.pdf]

LMU
KLINIKUM
DER UNIVERSITÄT MÜNCHEN

ARBEITSGRUPPE ARBEITS- UND
UMWELTEPIDEMIOLOGIE & NET TEACHING
LEITUNG: PROF. DR. KATJA RADON, MSC

INSTITUT UND POLIKLINIK FÜR
ARBEITS-, SOZIAL- U. UMWELTMEDIZIN
DIREKTOR: PROF. DR. MED. DENNIS NOWAK

CAMPUS INNENSTADT

Studien-ID: «ID2»

Fragebogen

SASKIA

Schichtarbeit, Schlafstörungen und kognitive Beeinträchtigungen im Alter

Studienleitung:

Dr. Tobias Weinmann, MSc
Institut und Poliklinik für Arbeits-, Sozial- und Umweltmedizin
Klinikum der Universität München (LMU)
Ziemssenstr. 1, 80336 München
Telefon: 089/4400-52372
Telefax: 089/4400-54954
E-Mail: tobias.weinmann@med.uni-muenchen.de

ALLGEMEINES

Wir möchten Sie zunächst um allgemeine Angaben zu Ihrer Person bitten.

1 **Wann wurden Sie geboren?**

I__I__I.I__I__I.19I__I__I
Tag Monat Jahr

2 **Sind Sie männlich oder weiblich?**

männlich ☐
weiblich ☐

3 **In welchem Land wurden Sie geboren?**

____

4 **Was ist Ihr Familienstand?**

ledig ☐
verheiratet/in Partnerschaft lebend ☐
geschieden ☐
verwitwet ☐

5 **Was ist Ihr höchster Schul- bzw. Hochschulabschluss?**

Hauptschulabschluss ☐
Realschulabschluss (Mittlere Reife) ☐
Fachhochschulreife (FOS, BOS, etc.) ☐
Abitur/Fachabitur ☐
Hochschule/Fachhochschule/Universität ☐
Kein Abschluss ☐
Anderer, ☐
welcher?

____

6 Was ist der höchster Schul- bzw. Hochschulabschluss Ihres <u>aktuellen bzw. letzten Partners</u>?

- Hauptschulabschluss .... ☐
- Realschulabschluss (Mittlere Reife) .... ☐
- Fachhochschulreife (FOS, BOS, etc.) .... ☐
- Abitur/Fachabitur .... ☐
- Hochschule/Fachhochschule/Universität .... ☐
- Kein Abschluss .... ☐
- Anderer, ... ☐
  welcher?

---

7 Welches ist Ihr höchster berufsbildender Abschluss?

- Fachschule/Berufsfachschule/Berufskolleg .... ☐
- Berufsschule/Lehre .... ☐
- Studium.. ☐
- Kein Abschluss.. ☐
- Anderer, ... ☐
  welcher?

---

8 Was ist Ihre aktuelle Tätigkeit?

- Vollzeit angestellt... ☐
- Teilzeit angestellt... ☐
- Rentner/in .... ☐
- Andere, ... ☐
  welche?

---

9 Haben Sie schon einmal ein Jahr lang geraucht?

„Ja" bedeutet mindestens 20 Päckchen Zigaretten oder 360g Tabak in Ihrem Leben oder ein Jahr lang eine Zigarette pro Tag oder eine Zigarre pro Woche

- Nein.. ☐ Bitte weiter mit **Frage 11**
- Ja .... ☐

10 Haben Sie innerhalb des letzten Monats geraucht?

Nein .... ☐

Ja .... ☐

11 Wie viel Bier, Wein und Schnaps haben Sie am letzten Wochenende, also am Samstag und Sonntag, getrunken?

Bier (auf **0,5 l** genau)	I___I___I,I___I Liter
Wein (auf **0,2 l** genau)	I___I___I, I___I Liter
Schnaps (**Anzahl der Gläser** à 0,02 l)	I___I___I Gläser

12 Wie viel Bier, Wein und Schnaps haben Sie am letzten Werktag getrunken?

Bier (auf **0,5 l** genau)	I___I___I, I___I Liter
Wein (auf **0,2 l** genau)	I___I___I, I___I Liter
Schnaps (**Anzahl der Gläser** à 0,02 l)	I___I___I Gläser

13 Wie häufig treiben Sie gewöhnlich Sport, so dass Sie außer Atem geraten oder schwitzen? Bitte nur eine Antwort!

Nie .... ☐

Weniger als einmal pro Monat .... ☐

Einmal pro Monat... ☐

Einmal pro Woche .... ☐

2 – 3 mal pro Woche .... ☐

4 – 6 mal pro Woche .... ☐

Jeden Tag .... ☐

14 Was ist Ihr aktuelles Gewicht?

I___I___I, I___I kg

15 Was ist Ihre Körpergröße?

I___I, I___I___I m

GESUNDHEIT

Nun möchten wir Sie zu Ihrer Gesundheit befragen. Wenn Sie sich bei einer der Fragen nicht ganz sicher sind, kreuzen Sie bitte „Nein“ an.

16 Haben Sie jemals an einer der folgenden Krankheiten oder Beschwerden gelitten?

16.1 Neurologische Probleme wie Migräne oder Epilepsie

Nein .... ☐

Ja .... ☐

Falls ja, welche?

____

16.2 Krebserkrankung

Nein .... ☐

Ja .... ☐

Falls ja, welche?

____

16.3 Psychiatrische Erkrankung wie Depression oder Angststörung

Nein .... ☐

Ja .... ☐

Falls ja, welche?

____

16.4 Neurodegenerative Erkrankung wie Alzheimer, Parkinson oder ALS

Nein .... ☐

Ja .... ☐

Falls ja, welche?

____

16.5 Probleme in den hormonellen und endokrinen Systemen wie Schilddrüsenkrankheit (Über-/Unterfunktion), Diabetes, etc.

Nein .... ☐

Ja .... ☐

Falls ja, welche?

____

16.6 Genetische Syndrome (Neurofibromatose, Turcot-Syndrom, etc.) oder angeborene Fehlbildungen

Nein .... ☐

Ja .... ☐

Falls ja, welche?

____

16.7 Allergien oder Asthma

Nein .... ☐

Ja .... ☐

Falls ja, welche?

____

16.8 Schlafstörungen (Einschlafstörungen, Durchschlafstörungen, vorzeitiges Erwachen, Schlafapnoe, etc.)

Nein .... ☐

Ja .... ☐

Falls ja, welche?

____

16.9 Andere schwerwiegende Krankheiten oder Beschwerden

Nein .... ☐

Ja .... ☐

Falls ja, welche?

____

SCHLAFVERHALTEN

Wir würden Sie nun gerne nach Ihrem **aktuellen Schlafverhalten an freien Tagen** sowie Ihrem Schlafverhalten an freien Tagen **als Sie 40 und 30 Jahre alt waren** fragen. Bitte füllen Sie die folgende Tabelle für jeden dieser drei Zeitpunkte aus.

17.1 bis 17.9	**Aktuell**	**Mit 40 Jahren**	**Mit 30 Jahren**
Ich gehe ins Bett um I__I__I:I__I__I Uhr.	I__I__I:I__I__I Uhrzeit	I__I__I:I__I__I Uhrzeit	I__I__I:I__I__I Uhrzeit
Manche Menschen bleiben noch eine Weile wach, wenn sie im Bett liegen! Ich bin bereit einzuschlafen um I__I__I:I__I__I Uhr.	I__I__I:I__I__I Uhrzeit	I__I__I:I__I__I Uhrzeit	I__I__I:I__I__I Uhrzeit
Um einzuschlafen brauche ich ________Minuten.	________ Minuten	________ Minuten	________ Minuten
Ich wache auf um I__I__I:I__I__I Uhr.	I__I__I:I__I__I Uhrzeit	I__I__I:I__I__I Uhrzeit	I__I__I:I__I__I Uhrzeit
Ich wache mit Wecker/ohne Wecker auf.	☐ Mit Wecker ☐ Ohne Wecker	☐ Mit Wecker ☐ Ohne Wecker	☐ Mit Wecker ☐ Ohne Wecker
Ich stehe auf nach ________Minuten.	________ Minuten	________ Minuten	________ Minuten
Ich mache tagsüber öfter ein Nickerchen	☐Ja ☐Nein	☐Ja ☐Nein	☐Ja ☐Nein
Wenn ja: Ich mache das Nickerchen zwischen I__I__I:I__I__I und I__I__I:I__I__I Uhr.	Von I__I__I:I__I__I bis I__I__I:I__I__I Uhr.	Von I__I__I:I__I__I bis I__I__I:I__I__I Uhr.	Von I__I__I:I__I__I bis I__I__I:I__I__I Uhr.
Oft hört man von „Morgen-Typen“ und „Abend-Typen“. Wie schätzen Sie sich selbst ein?	☐ Definitiv ein Morgen-Typ ☐ Eher ein Morgen-Typ ☐ Eher ein Abend-Typ ☐ Definitiv ein Abend-Typ	☐ Definitiv ein Morgen-Typ ☐ Eher ein Morgen-Typ ☐ Eher ein Abend-Typ ☐ Definitiv ein Abend-Typ	☐ Definitiv ein Morgen-Typ ☐ Eher ein Morgen-Typ ☐ Eher ein Abend-Typ ☐ Definitiv ein Abend-Typ

BERUFSGESCHICHTE

Wir möchten Sie des Weiteren nach **<u>allen hauptberuflichen Tätigkeiten</u>** fragen, die Sie bisher hatten. Mit Hauptberuf meinen wir die Tätigkeit, mit der Sie die meiste Zeit verbracht haben. Bitte berücksichtigen Sie alle Tätigkeiten, die Sie **mindestens 12 Monate** lang ausgeübt haben. Beginnen Sie bitte mit Ihrer aktuellen bzw. zuletzt ausgeführten beruflichenTätigkeit.

18 Erste (=aktuelle/letzte) Tätigkeit

18.1 Startdatum: |__|__|__|__|/|__|__|
Jahr Monat

Enddatum: |__|__|__|__|/|__|__|
Jahr Monat
(falls Sie immer noch in diesem Job arbeiten, geben Sie bitte das aktuelle D Datum an)

18.2 Wie lautet der Name der Einrichtung/des Unternehmens?

____

18.3 Was ist/war Ihre Tätigkeit?

____

18.4 Haben Sie Vollzeit, Teilzeit oder gelegentlich in diesem Job gearbeitet?
|1| Vollzeit
|2| Teilzeit
|3| Gelegentlich

18.5 Hat diese Tätigkeit <u>Nachtschichten</u>, d.h. Schichten, die den Zeitraum zwischen 0 und 5 Uhr einschließen, beinhaltet?
Nein...☐ Bitte weiter mit **Frage 18.7**
Ja ....☐

18.6 Wenn ja, wie viele <u>Nachtschichten</u> sind/waren es durchschnittlich im Monat?

Anzahl der Nachtschichten:___________

18.7 Hat diese Tätigkeit <u>Frühschichten</u>, d.h. Schichten mit Dienstbeginn vor 7 Uhr, beinhaltet?
Nein...☐ Bitte weiter mit **Frage 19**
Ja ....☐

18.8 Wenn ja, wie viele <u>Frühschichten</u> sind/waren es durchschnittlich im Monat?

Anzahl der Frühschichten:___________

19 Zweite Tätigkeit

19.1 Startdatum: |__|__|__|__|/|__|__|
Jahr Monat

Enddatum: |__|__|__|__|/|__|__|
Jahr Monat
(falls Sie immer noch in diesem Job arbeiten, geben Sie bitte das aktuelle Datum an)

19.2 Wie lautet der Name der Einrichtung/des Unternehmens?

___

19.3 Was ist/war Ihre Tätigkeit?

___

19.4 Haben Sie Vollzeit, Teilzeit oder gelegentlich in diesem Job gearbeitet?
|1| Vollzeit
|2| Teilzeit
|3| Gelegentlich

19.5 Hat diese Tätigkeit Nachtschichten, d.h. Schichten, die den Zeitraum zwischen 0 und 5 Uhr einschließen, beinhaltet?
Nein..☐ Bitte weiter mit **Frage 19.7**
Ja ....☐

19.6 Wenn ja, wie viele Nachtschichten sind/waren es durchschnittlich im Monat?

Anzahl der Nachtschichten:___________

19.7 Hat diese Tätigkeit Frühschichten, d.h. Schichten mit Dienstbeginn vor 7 Uhr, beinhaltet?
Nein..☐ Bitte weiter mit **Frage 20**
Ja ....☐

19.8 Wenn ja, wie viele Frühschichten sind/waren es durchschnittlich im Monat?

Anzahl der Frühschichten:___________

20 Dritte Tätigkeit

20.1 Startdatum: |__|__|__|__|/|__|__|
Jahr Monat

Enddatum: |__|__|__|__|/|__|__|
Jahr Monat
(falls Sie immer noch in diesem Job arbeiten, geben Sie bitte das aktuelle Datum an)

20.2 Wie lautet der Name der Einrichtung/des Unternehmens?

___

20.3 Was ist/war Ihre Tätigkeit?

___

20.4 Haben Sie Vollzeit, Teilzeit oder gelegentlich in diesem Job gearbeitet?
|1| Vollzeit
|2| Teilzeit
|3| Gelegentlich

20.5 Hat diese Tätigkeit <u>Nachtschichten</u>, d.h. Schichten, die den Zeitraum zwischen 0 und 5 Uhr einschließen, beinhaltet?
Nein..☐ Bitte weiter mit **Frage 20.7**
Ja ....☐

20.6 Wenn ja, wie viele <u>Nachtschichten</u> sind/waren es durchschnittlich im Monat?

Anzahl der Nachtschichten:___________

20.7 Hat diese Tätigkeit <u>Frühschichten</u>, d.h. Schichten mit Dienstbeginn vor 7 Uhr, beinhaltet?
Nein..☐ Bitte weiter mit **Frage 21**
Ja ....☐

20.8 Wenn ja, wie viele <u>Frühschichten</u> sind/waren es durchschnittlich im Monat?

Anzahl der Frühschichten:___________

21 Vierte Tätigkeit

21.1 Startdatum: |__|__|__|__|/|__|__|
Jahr Monat

Enddatum: |__|__|__|__|/|__|__|
Jahr Monat

(falls Sie immer noch in diesem Job arbeiten, geben Sie bitte das aktuelle Datum an)

21.2 Wie lautet der Name der Einrichtung/des Unternehmens?

___

21.3 Was ist/war Ihre Tätigkeit?

___

21.4 Haben Sie Vollzeit, Teilzeit oder gelegentlich in diesem Job gearbeitet?

|1| Vollzeit
|2| Teilzeit
|3| Gelegentlich

21.5 Hat diese Tätigkeit <u>Nachtschichten</u>, d.h. Schichten, die den Zeitraum zwischen 0 und 5 Uhr einschließen, beinhaltet?

Nein .... ☐ Bitte weiter mit **Frage 21.7**

Ja .... ☐

21.6 Wenn ja, wie viele <u>Nachtschichten</u> sind/waren es durchschnittlich im Monat?

Anzahl der Nachtschichten:___________

21.7 Hat diese Tätigkeit <u>Frühschichten</u>, d.h. Schichten, die den Zeitraum zwischen 0 und 5 Uhr einschließen, beinhaltet?

Nein .... ☐ Bitte weiter mit **Frage 22**

Ja .... ☐

21.8 Wenn ja, wie viele <u>Frühschichten</u> sind/waren es durchschnittlich im Monat?

Anzahl der Frühschichten:___________

22 Fünfte Tätigkeit

22.1 Startdatum: |__|__|__|__|/|__|__|
Jahr Monat

Enddatum: |__|__|__|__|/|__|__|
Jahr Monat
(falls Sie immer noch in diesem Job arbeiten, geben Sie bitte das aktuelle Datum an)

22.2 Wie lautet der Name der Einrichtung/des Unternehmens?

____

22.3 Was ist/war Ihre Tätigkeit?

____

22.4 Haben Sie Vollzeit, Teilzeit oder gelegentlich in diesem Job gearbeitet?
|1| Vollzeit
|2| Teilzeit
|3| Gelegentlich

22.5 Hat diese Tätigkeit Nachtschichten, d.h. Schichten, die den Zeitraum zwischen 0 und 5 Uhr einschließen, beinhaltet?
Nein .... ☐ Bitte weiter mit **Frage 22.7**
Ja .... ☐

22.6 Wenn ja, wie viele Nachtschichten sind/waren es durchschnittlich im Monat?
Anzahl der Nachtschichten:___________

22.7 Hat diese Tätigkeit Frühschichten, d.h. Schichten mit Dienstbeginn vor 7 Uhr, beinhaltet?
Nein .... ☐ Bitte weiter mit **Frage 23**
Ja .... ☐

22.8 Wenn ja, wie viele Frühschichten sind/waren es durchschnittlich im Monat?
Anzahl der Frühschichten:___________

Falls Sie weitere Tätigkeiten hatten, geben Sie diese bitte auf der Rückseite der letzten Seite des Fragebogens an.

SCHICHTARBEIT

Abschließend möchten wir Sie um einige weitere Angaben zu Ihrer Arbeit in Schichtsystemen fragen. Berücksichtigen Sie hierbei bitte **alle beruflichen Tätigkeiten**, die Sie jemals ausgeübt haben. Wenn Sie sich bei einer der Fragen nicht ganz sicher sind, kreuzen Sie bitte „Nein" an.

23 Haben Sie jemals in Schichtarbeit gearbeitet?

Schichtarbeit heißt, dass Beginn oder Ende der Tätigkeit **regelmäßig** außerhalb der als üblich betrachteten Tagesarbeitszeit (von 7 bis 18 Uhr) liegen

Nein .... ☐

Ja .... ☐

24 Falls ja, wie viele Jahre haben Sie insgesamt in Ihrem Leben in Schichtarbeit gearbeitet?

(wenn weniger als 1 Jahr bitte 0 eintragen)

Anzahl der Jahre:___________

25 Wie viele Jahre haben Sie insgesamt mit Nachtschichten, d.h. mit Schichten die den Zeitraum zwischen 0 und 5 Uhr einschließen, gearbeitet?

(wenn weniger als 1 Jahr bitte 0 eintragen)

Anzahl der Jahre:___________

26 Wie viele Jahre haben Sie insgesamt ausschließlich in Nachtschichten gearbeitet? (d.h. keine Schichten außer Nachtschicht)

(wenn weniger als 1 Jahr bitte 0 eintragen)

Anzahl der Jahre:___________

27 Wann haben Sie zuletzt in Nachtschicht gearbeitet?

I__I__I__I__I
Jahr

28 Wie viele Jahre haben Sie insgesamt mit Frühschichten, d.h. mit Dienstbeginn vor 7 Uhr, gearbeitet?

(wenn weniger als 1 Jahr bitte 0 eintragen)

Anzahl der Jahre:___________

29 Wie viele Jahre haben Sie insgesamt ausschließlich in Frühschichten gearbeitet? (d.h. keine Schichten außer Frühschicht)

(wenn weniger als 1 Jahr bitte 0 eintragen)

Anzahl der Jahre:___________

30 Wann haben Sie zuletzt in Frühschicht gearbeitet?

I__I__I__I__I
Jahr

Herzlichen Dank für Ihre Teilnahme!

Haben Sie noch Anmerkungen zu diesem Fragebogen?

Für Anregungen sind wir dankbar!
