# Supplementary material for: Shift work and cognitive impairment in later life – results of a cross-sectional pilot study testing the feasibility of a large-scale epidemiologic investigation
Source: BMC Public Health. 2018 Nov 14;18:1256. doi: 10.1186/s12889-018-6171-5 (PMC6234599; doi:10.1186/s12889-018-6171-5)
Supplement: Supplementary file 2 — English translated version of the study questionnaire. (PDF 113 kb) [file 12889_2018_6171_MOESM2_ESM.pdf]

LMU
KLINIKUM
DER UNIVERSITÄT MÜNCHEN
ARBEITSGRUPPE ARBEITS- UND UMWELTEPIDEMIOLOGIE & NET TEACHING
LEITUNG: PROF. DR. KATJA RADON, MSC
INSTITUT UND POLIKLINIK FÜR ARBEITS-, SOZIAL- U. UMWELTMEDIZIN
DIREKTOR: PROF. DR. MED. DENNIS NOWAK
CAMPUS INNENSTADT

Study ID:

Questionnaire

SASKIA

Shift work, sleep disturbances and cognitive impairment in later life

Study coordinator:
Dr. Tobias Weinmann, MSc
Institut und Poliklinik für Arbeits-, Sozial- und Umweltmedizin
Klinikum der Universität München (LMU)
Ziemssenstr. 1, 80336 München
Telefon: 089/4400-52372
Telefax: 089/4400-54954
E-Mail: tobias.weinmann@med.uni-muenchen.de

GENERAL INFORMATION

First, we would like to ask for general information regarding your person.

1 What is your birth date?

I__I__I.I__I__I.19I__I__I
Day Month Year

2 Are you male or female?

Male .... ☐
Female .... ☐

3 Which is your birth country?

____

4 What is your marital status?

Single .... ☐
Married/living in partnership .... ☐
Divorced .... ☐
Widowed .... ☐

5 What is your highest educational qualification?

Secondary modern school qualification .... ☐
High-school diploma .... ☐
University of applied sciences entrance qualification .... ☐
A level / Advanced Vocational Certificate of Education .... ☐
University degree .... ☐
No qualification .... ☐
Other, ... ☐
which one?

____

6 What is your highest educational qualification <u>of your current / last partner</u>?

- Secondary modern school qualification ☐
- High-school diploma ☐
- University of applied sciences entrance qualification ☐
- A level / Advanced Vocational Certificate of Education ☐
- University degree ☐
- No qualification ☐
- Other, ☐
  which one?

____

7 What is your highest occupational training?

- Professional school ☐
- Vocational/apprenticeship ☐
- University degree ☐
- No degree ☐
- Other, ☐
  which one?

____

8 What is your current occupation?

- Full-time ☐
- Part-time ☐
- Pensioner ☐
- Other, ☐
  which one?

____

9 Did you ever smoke for at least one year?

„Yes" means at least 20 packs of cigarettes or 360g of tobacco in your life or one cigarette per day or one cigar per week for one year

No.. ☐ Please go to **question 11**

Yes.. ☐

10 Did you smoke within the last month?

No.. ☐

Yes.. ☐

11 How much beer, wine or liquor did you drink <u>during the last weekend</u>, i.e. on Saturday <u>and</u> Sunday?

Beer (with an accuracy of 0,5 l) I___I___I,I___I liters

Wine (with an accuracy of 0,2 l) I___I___I, I___I liters

Liquor (Number of glasses of 0,02 l) I___I___I glasses

12 How much beer, wine or liquor did you drink <u>during the last weekday?</u>

Beer (with an accuracy of 0,5 l) I___I___I,I___I liters

Wine (with an accuracy of 0,2 l) I___I___I, I___I liters

Liquor (Number of glasses of 0,02 l) I___I___I glasses

13 How oft do you exercise, so that you sweat or get out of breath? Please tick only one box!

Never.. ☐

Less than once per month .... ☐

Once per month.. ☐

Once per week .... ☐

2 – 3 times per week .... ☐

4 – 6 times per week .... ☐

Every day.. ☐

14 What is your current weight?

I___I___I, I___I kg

15 What is your current height?

I___I, I___I___I m

HEALTH

Now we would like to ask your some question regarding your health. If you are not sure how to answer one of the questions, please indicate “no”.

16 Did your ever suffer from one of the following diseases or conditions?

16.1 Neurologic conditions such as migraine or epilepsy

No.. ☐

Yes.. ☐

If yes, which ones?

____

16.2 Cancer

No.. ☐

Yes.. ☐

If yes, which ones?

____

16.3 Psychiatric diseases such as depression or anxiety disorder

No.. ☐

Yes.. ☐

If yes, which ones?

____

16.4 Neurodegenerative diseases such as Alzheimer‘s, Parkinson’s or ALS

No.. ☐

Yes.. ☐

If yes, which ones?

____

16.5 Problems in the hormonal and endocrine systems such as thyroid disease (hyperthyroidism/hypothyroidism), diabetes, etc.

No..☐

Yes..☐

If yes, which ones?

16.6 Genetic syndromes (Neurofibromatosis, Turcot syndrome, etc.) or congenital malformations

No..☐

Yes..☐

If yes, which ones?

16.7 Allergies or asthma

No..☐

Yes..☐

If yes, which ones?

16.8 Sleep disturbances (diffculty to fall asleep, diffculty to sleep through , early awakening, sleep apnoea, etc.)

No..☐

Yes..☐

If yes, which ones?

16.9 Other major dieseases or conditions

No..☐

Yes..☐

If yes, which ones?

SLEEPING BEHAVIOUR

We would now like to ask you about your **current sleeping behaviour on free days** and your sleeping behavior on free days **when you were 40 and 30 years old**. Please complete the following for each of those three time points.

17.1 to 17.9	Currently	With 40 years	With 30 years
I go to bed at I__I__I:I__I__I o'clock.	I__I__I:I__I__I Time	I__I__I:I__I__I Time	I__I__I:I__I__I Time
Note that some people stay awake for some time when in bed! I actually get ready to fall asleep at I__I__I:I__I__I o'clock.	I__I__I:I__I__I Time	I__I__I:I__I__I Time	I__I__I:I__I__I Time
I need ________minutes to fall asleep.	________ Minutes	________ Minutes	________ Minutes
I wake up at I__I__I:I__I__I o'clock.	I__I__I:I__I__I Time	I__I__I:I__I__I Time	I__I__I:I__I__I Time
I wake up with/without an alarm clock.	☐ With alarm ☐ Without alarm	☐ With alarm ☐ Without alarm	☐ With alarm ☐ Without alarm
After ________minutes I get up.	________ Minutes	________ Minutes	________ Minutes
Once in a while I take a nap during the day	☐Yes ☐No	☐Yes ☐No	☐Yes ☐No
If yes: I take the nap from I__I__I:I__I__I to I__I__I:I__I__I o'clock.	From I__I__I:I__I__I to I__I__I:I__I__I	From I__I__I:I__I__I to I__I__I:I__I__I	From I__I__I:I__I__I to I__I__I:I__I__I
Often one hears about "morning-types" and "evening-types." Which one of these types do you consider yourself to be?	☐ Definitive morning-type ☐ Rather morning-type ☐ Rather evening-type ☐ Definitive evening-type	☐ Definitive morning-type ☐ Rather morning-type ☐ Rather evening-type ☐ Definitive evening-type	☐ Definitive morning-type ☐ Rather morning-type ☐ Rather evening-type ☐ Definitive evening-type

OCCUPATIONAL HISTORY

Now we would like to ask for **all main occupations** that you had so far. With main occupation we mean all jobs with which you spent the most of your time. Pleae consider all occupations that you carried out for **at least 12 months.** Please start with your current/last occupation.

18 First (=current/last) job

18.1 Start date: |__|__|__|__|/|__|__|
Year Month

End date: |__|__|__|__|/|__|__|
Year Month

(if you still work in this job, please indicate the current date)

18.2 What is the name of the institution/company?

____

18.3 What is/was your task?

____

18.4 Do/Did you work full-time, part-time or occasionally in this job?

|1| Full-time
|2| Part-time
|3| Occasionally

18.5 Did this job include night shifts, i.e., shifts that included the time period between 00:00 am and 05:00 am?

No..☐ Please go to **question 18.7**

Yes...☐

18.6 If yes, how many night shifts do/did you work on average per month?

Number of night shifts:___________

18.7 Did this job include morning shifts, i.e., shifts that started before 07:00 am?

No..☐ Please go to **question 19**

Yes...☐

18.8 If yes, how many morning shifts do/did you work on average per month?

Number of morning shifts:___________

19 Second Job

19.1 Start date: |__|__|__|__|/|__|__|
Year Month

End date: |__|__|__|__|/|__|__|
Year Month
(if you still work in this job, please indicate the current date)

19.2 What is the name of the institution/company?

____

19.3 What is/was your task?

____

19.4 Did you work full-time, part-time or occasionally in this job?
|1| Full-time
|2| Part-time
|3| Occasionally

19.5 Did this job include <u>night shifts</u>, i.e., shifts that included the time period between 00:00 am and 05:00 am?
No..☐ Please go to **question 19.7**
Yes..☐

19.6 If yes, how many <u>night shifts do/did you work</u> on average per month?
Number of night shifts:___________

19.7 Did this job include <u>morning shifts</u>, i.e., shifts that started before 07:00 am?
No..☐ Please go to **question 20**
Yes..☐

19.8 If yes, how many <u>morning shifts do/did you work</u> on average per month?
Number of morning shifts:___________

20 Third job

20.1 Start date: |__|__|__|__|/|__|__|
Year Month

End date: |__|__|__|__|/|__|__|
Year Month
(if you still work in this job, please indicate the current date)

20.2 What is the name of the institution/company?

____

20.3 What is/was your task?

____

20.4 Did you work full-time, part-time or occasionally in this job?
|1| Full-time
|2| Part-time
|3| Occasionally

20.5 Did this job include <u>night shifts</u>, i.e., shifts that included the time period between 00:00 am and 05:00 am?
No...☐ Please go to **question 20.7**
Yes...☐

20.6 If yes, how many <u>night shifts do/did you work</u> on average per month?
Number of night shifts:__________

20.7 Did this job include <u>morning shifts</u>, i.e., shifts that started before 07:00 am?
No...☐ Please go to **question 21**
Yes...☐

20.8 If yes, how many <u>morning shifts do/did you work</u> on average per month?
Number of morning shifts:__________

21 Fourth job

21.1 Start date: I__I__I__I__I/I__I__I
Year Month

End date: I__I__I__I__I/I__I__I
Year Month
(if you still work in this job, please indicate the current date)

21.2 What is the name of the institution/company?

____

21.3 What is/was your task?

____

21.4 Did you work full-time, part-time or occasionally in this job?
|1| Full-time
|2| Part-time
|3| Occasionally

21.5 Did this job include <u>night shifts</u>, i.e., shifts that included the time period between 00:00 am and 05:00 am?
No...☐ Please go to **question 21.7**
Yes..☐

21.6 If yes, how many <u>night shifts do/did you work</u> on average per month?
Number of night shifts:___________

21.7 Did this job include <u>morning shifts</u>, i.e., shifts that started before 07:00 am?
No...☐ Please go to **question 22**
Yes..☐

21.8 If yes, how many <u>morning shifts do/did you work</u> on average per month?
Number of morning shifts:___________

22 Fifth job

22.1 Start date: |__|__|__|__|/|__|__|
Year Month

End date: |__|__|__|__|/|__|__|
Year Month
(if you still work in this job, please indicate the current date)

22.2 What is the name of the institution/company?

____

22.3 What is/was your task?

____

22.4 Did you work full-time, part-time or occasionally in this job?
|1| Full-time
|2| Part-time
|3| Occasionally

22.5 Did this job include <u>night shifts</u>, i.e., shifts that included the time period between 00:00 am and 05:00 am?
No..☐ Please go to **question 22.7**
Yes..☐

22.6 If yes, how many <u>night shifts do/did you work</u> on average per month?
Number of night shifts:___________

22.7 Did this job include <u>morning shifts</u>, i.e., shifts that started before 07:00 am?
No..☐ Please go to **question 23**
Yes..☐

22.8 If yes, how many <u>morning shifts do/did you work</u> on average per month?
Number of morning shifts:___________

If you had additional jobs, please indicate them on the last page of the questionnaire.

SHIFT WORK

Lastly, we would like to ask you for some information regarding your work in **shift systems**. Please consider all **jobs** that you ever had. If you are not sure how to answer one of the questions, please indicate "no".

23 Did you ever work in shifts?

Working in shift means that start or end of the job were **regularly** outside usual working hours (from 07:00 am to 18:00 pm).

No.. ☐

Yes.. ☐

24 If yes, how many years did you work in shifts in total during your life?

(If less than 1 year please indicate 0)

Number of years:___________

25 How many years in total did you work in night shifts, i.e., shifts that include the time period between 00:00 am and 05:00 am?

(If less than 1 year please indicate 0)

Number of years:___________

26 How many years in total did you work solely in night shifts?

(i.e., no shifts but night shifts)

(If less than 1 year please indicate 0)

Number of years:___________

27 When was the last time that you worked in night shifts?

I__I__I__I__I
Year

28 How many years in total did you work in morning shifts, i.e., starting bevor 07:00 am?

(If less than 1 year please indicate 0)

Number of years:___________

29 How many years in total did you work solely in morning shifts?

(i.e., no shifts but morning shifts)

(If less than 1 year please indicate 0)

Number of years:___________

30 When was the last time that you worked in morning shifts?

|__|__|__|__|
Year

Thank you very much for your participation!

Do you have comments to this questionnaire?

We are happy to year your suggestions!

____

____
